# Infectious origin of Alzheimer’s disease: Amyloid beta as a component of brain antimicrobial immunity

**DOI:** 10.1371/journal.ppat.1010929

**Published:** 2022-11-17

**Authors:** Iveta Vojtechova, Tomas Machacek, Zdenka Kristofikova, Ales Stuchlik, Tomas Petrasek

**Affiliations:** 1 National Institute of Mental Health, Klecany, Czech Republic; 2 Laboratory of Neurophysiology of Memory, Institute of Physiology of the Czech Academy of Sciences, Prague, Czech Republic; 3 Department of Parasitology, Faculty of Science, Charles University, Prague, Czech Republic; University of North Carolina at Chapel Hill School of Medicine, UNITED STATES

## Abstract

The amyloid cascade hypothesis, focusing on pathological proteins aggregation, has so far failed to uncover the root cause of Alzheimer’s disease (AD), or to provide an effective therapy. This traditional paradigm essentially explains a *mechanism* involved in the development of sporadic AD rather than its *cause*. The failure of an overwhelming majority of clinical studies (99.6%) demonstrates that a breakthrough in therapy would be difficult if not impossible without understanding the etiology of AD. It becomes more and more apparent that the AD pathology might originate from brain infection. In this review, we discuss a potential role of bacteria, viruses, fungi, and eukaryotic parasites as triggers of AD pathology. We show evidence from the current literature that amyloid beta, traditionally viewed as pathological, actually acts as an antimicrobial peptide, protecting the brain against pathogens. However, in case of a prolonged or excessive activation of a senescent immune system, amyloid beta accumulation and aggregation becomes damaging and supports runaway neurodegenerative processes in AD. This is paralleled by the recent study by Alam and colleagues (2022) who showed that alpha-synuclein, the protein accumulating in synucleinopathies, also plays a critical physiological role in immune reactions and inflammation, showing an unforeseen link between the 2 unrelated classes of neurodegenerative disorders. The multiplication of the *amyloid precursor protein* gene, recently described by Lee and collegues (2018), and possible reactivation of human endogenous retroviruses by pathogens fits well into the same picture. We discuss these new findings from the viewpoint of the infection hypothesis of AD and offer suggestions for future research.

## Author summary

More than a century after its discovery, Alzheimer’s disease (AD) remains incurable and mysterious. The dominant hypothesis of amyloid cascade has succeeded in explaining the key pathological mechanism, but not its trigger. Amyloid beta has been traditionally considered a pathological peptide, and its physiological functions remain poorly known. These knowledge gaps have contributed to repeated failures of clinical studies. The emerging infectious hypothesis of AD considers central nervous system (CNS) infection the primary trigger of sporadic AD. A closely connected hypothesis claims that amyloid beta is an antimicrobial peptide. In this review, we discuss the available evidence for the involvement of infections in AD, coming from epidemiological studies, *post mortem* analyses of brain tissue, and experiments *in vitro* and *in vivo*. We argue there is no unique “Alzheimer’s germ,” instead, AD is a general reaction of the CNS to chronic infections, in the milieu of an aged immune system. The pathology may become self-sustained even without continuous presence of microbes in the brain. Importantly, the infectious hypothesis leads to testable predictions. Targeting amyloid beta should be ineffective, unless the triggering pathogen and inflammatory response are addressed as well. Meticulous control of selected infections might be the best near-term strategy for AD prevention.

## Introduction

Alzheimer’s disease (AD) belongs among the most feared diseases in the developed world. The available treatment is unable to affect the disease progress efficiently and offers only minor and temporary alleviation of some symptoms [1]. Search for novel therapeutics is ongoing, but no less than 413 trials have failed during 2002 to 2012 [2] and the drug failure rate has reached 99.6% [3–5]. It is the poorly known AD etiology that precludes causal treatment. The leading amyloid cascade hypothesis has provided many important insights and is supported by an impressive amount of experimental data, but it essentially explains an involved *mechanism* rather than *the cause* of the disease development. We propose that taking one step up the causal chain might be necessary for understanding the true nature of the disease. The main goal of this review is to explore the hypothesis of the essential role of pathogens and immune system activation in AD development and a protective role of amyloid beta peptide as a part of brain immunity. This might open the door to new approaches beyond the traditional paradigm and hopefully also new therapeutic strategies.

## Alzheimer’s disease: Hallmarks, forms, and the amyloid cascade hypothesis

**Fig 1:**
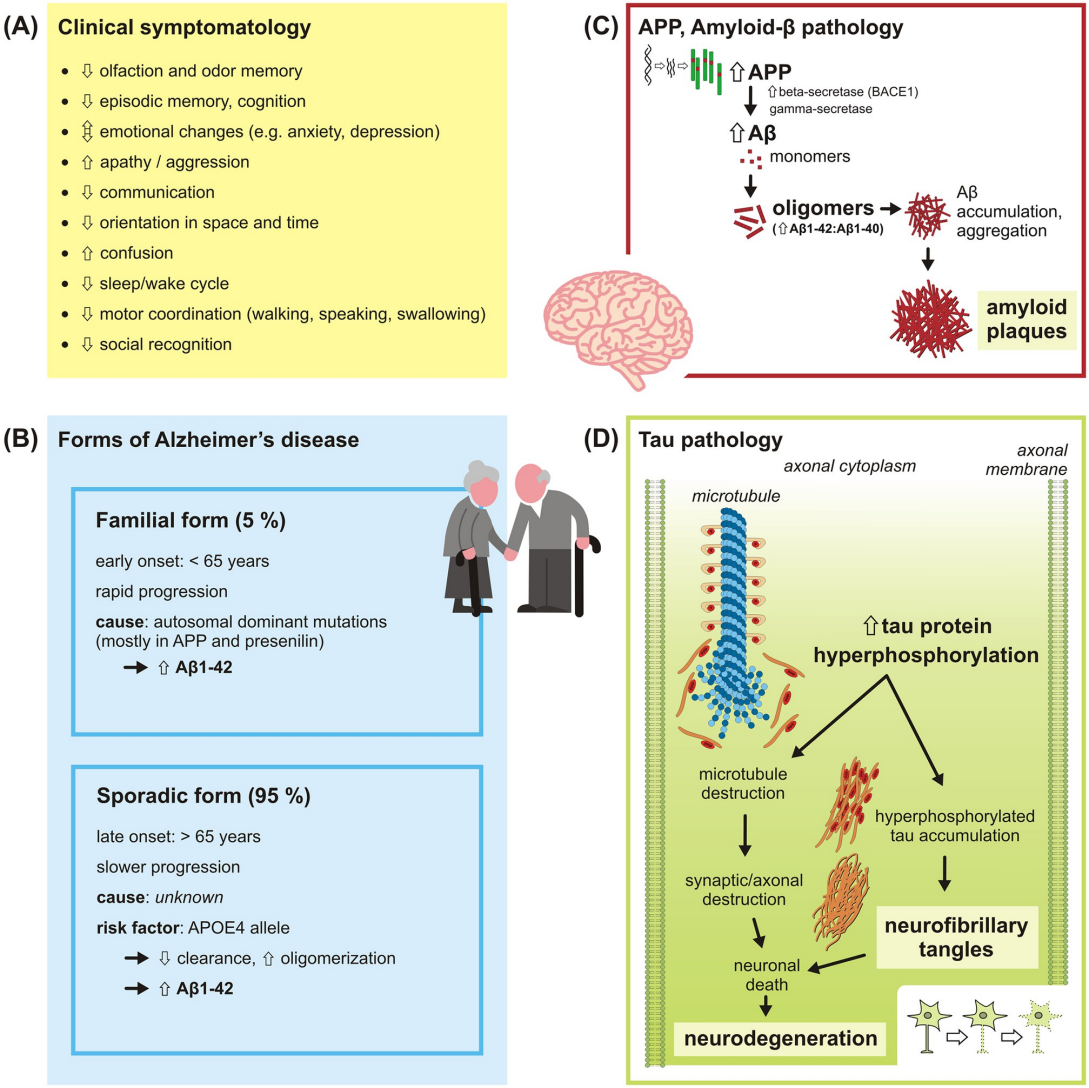
AD: Clinical symptomatology, forms, and Aβ and tau protein pathologies. (A) AD clinical symptomatology. The “down” arrows represent impairment or disruption of the brain functions in AD patients while “up” arrows symbolize the increase. (B) Two AD forms, familial and sporadic, differing in time of the onset, progression rate, and a cause, can be recognized. The familial form is associated with autosomal dominant mutations leading to inherently increased production of Aβ, especially the highly fibrillogenic Aβ ending at residue 42, (Aβ1–42). By contrast, the sporadic form does not follow mendelian inheritance and its causative triggers remain unknown. The strongest known genetic factor is APOE4. (C) In AD, the monomers of Aβ, which are cleavage fragments of APP, aggregate into oligomers. The oligomers, especially the cytoplasmic ones, are believed to be the most toxic, although extracellular amyloid plaques are the most conspicuous outcome of the process. (D) The tau protein normally binds to and stabilizes microtubules within neuronal axons. In an AD patient brain, tau protein is hyperphosphorylated and accumulates into neurofibrillary tangles, which leads to destabilization of cytoskeleton, disruption of synapses, and finally death of neurons. Aβ, amyloid beta; AD, Alzheimer’s disease; APOE4, apolipoprotein E4 allele; APP, amyloid precursor protein; BACE1, beta-site APP-cleaving enzyme.

AD is a neurodegenerative disease with inconspicuous onset and usually slow progression, typically affecting the elderly. The clinical stage of the disease is preceded by a mild cognitive impairment (MCI), a cognitive decline more severe than expected at a particular age [6]. Then, AD clinical symptomatology starts with impairment of olfaction and odor memory [7], episodic memory and disruption of cognitive functions, emotional changes, and apathy or aggression. Later, impaired communication, disorientation, confusion, disrupted sleep–wake cycle, motor coordination deficit appear and in the late phase, patients suffer from disruption of social recognition including the family and caregivers, and overall personality transformation (Fig 1A) [8]. However, the pathophysiological processes start many years before the clinical symptoms are manifested [6].

Two AD forms with similar symptomatology, but different etiology, can be recognized (Fig 1B). The familial form is characterized by early onset (<65 years) and rapid progression. It is associated with mutations leading to increased production of amyloid beta (Aβ, see below), especially the highly fibrillogenic Aβ ending at residue 42, (Aβ1–42; [9]). By contrast, the sporadic form has late onset (>65 years) and unknown causative triggers. While identified familial genetic mutations in amyloid precursor protein (APP) and presenilin [10–12], a component of gamma-secretase complex [13], are responsible for only about 1% of all AD cases, the sporadic form afflicts more than 95% of the patients [14], which is, however, not much reflected in animal AD models research. The strongest known genetic risk factor in the sporadic form is apolipoprotein E4 allele (APOE4) [15,16]. Although the knowledge about an involvement of APOE4 in AD pathology is rather poor, several hypotheses consider affecting Aβ clearance [17] and oligomerization [18].

Aβ peptide is one of the key molecules in the AD pathology. The monomers of Aβ are cleavage fragments of APP produced by the beta-secretase (beta-site APP-cleaving enzyme, BACE1) [10–12] and gamma-secretase [13]. Aβ occurs in both intra- and extracellular space and is prone to aggregation. In AD, Aβ aggregates into extracellular amyloid plaques that are the most conspicuous outcome of the process (Fig 1C). However, it is the oligomers that are the most toxic and contribute the most to AD progress [19–21]. Traditionally, Aβ peptide was believed to be the key cause of AD pathology, and considered only a byproduct of APP catabolism, lacking a normal physiological function. Later, it was found that Aβ is produced also under normal conditions [22] and plays an important role in many physiological processes, including response to cerebral infection [23]. Importance of Aβ is evidenced by its presence in all vertebrates, with high degree of sequence homology [14,23].

The tau protein is the second important molecule in AD pathology [24], normally binding to and stabilizing microtubules within axons. In an AD patient brain, tau protein is hyperphosphorylated, which leads to unbinding from microtubules and destabilization of cytoskeleton, contributing to final neuronal loss through synapse disruption (Fig 1D) [25]. Hyperphosphorylated tau is accumulated into neurofibrillary tangles intracellularly and later also outside the neurons [26]. It seems that both pathologies (Aβ overproduction and tau hyperphosphorylation) might influence or potentiate each other, and both contribute to synapse disruption leading to neurodegeneration [27,28]. Importantly, the presence of both pathologies is the necessary criterion for AD diagnosis [6]. Nevertheless, it is probable that the onset of AD requires a certain (not yet identified) trigger event, initiating the process of aberrant protein accumulation through a chain reaction or positive feedback.

The amyloid cascade hypothesis supposes that the deposition of Aβ peptide in the brain is the main cause of AD pathology [29,30]. In our view, the hypothesis offers a good description of the process of amyloid accumulation and the resulting pathology. However, it mostly does not consider the physiological roles of Aβ and is not concerned with the factors that initiate its (over)production in sporadic AD.

Beyond Aβ and tau pathologies, neuroinflammation has emerged as the third vital contributor to AD pathogenesis. Microglia are the key innate immune players in the central nervous system (CNS), and they can recognize and remove excessive Aβ deposits [31]. However, prolonged microglia activation triggers an inflammatory cascade leading to neuronal damage [32]. This might initiate hyperphosphorylation of tau protein that promotes AD pathology [33,34]. On the other hand, microglia lose their scavenging capacity in aging brains, which leads to reduced removal of Aβ deposits, facilitating AD pathology anyway [35,36]. Altogether, it demonstrates that neuroinflammation can enhance ongoing AD pathology. Moreover, Lee and colleagues demonstrated that systemic inflammation induced by bacterial endotoxin affects the pathological processing of APP [37]. The mechanism is presumably mediated by tumor necrosis factor (TNF) and interferon-gamma [38,39], pro-inflammatory cytokines commonly produced by activated innate immune cells. These data indicate that the pro-inflammatory milieu can initiate AD pathology via alteration of APP/Aβ homeostasis.

Another evidence showing that neuroinflammation plays an essential role in AD development, pushing the Aβ pathology to the sidelines, could be the existence of “resilient” individuals. In fact, some portion of individuals with high burden of amyloid plaques and tau tangles in the brain do not develop dementia. The reasons for such brain “resilience” have not been clarified so far. However, the lack of neuroinflammation represents one suggestion as the activated microglia and astroglia, present around synapses and accompanied by the release of pro-inflammatory cytokines, are typical only for AD brains, but not for the resilient brains [40].

**Fig 2:**
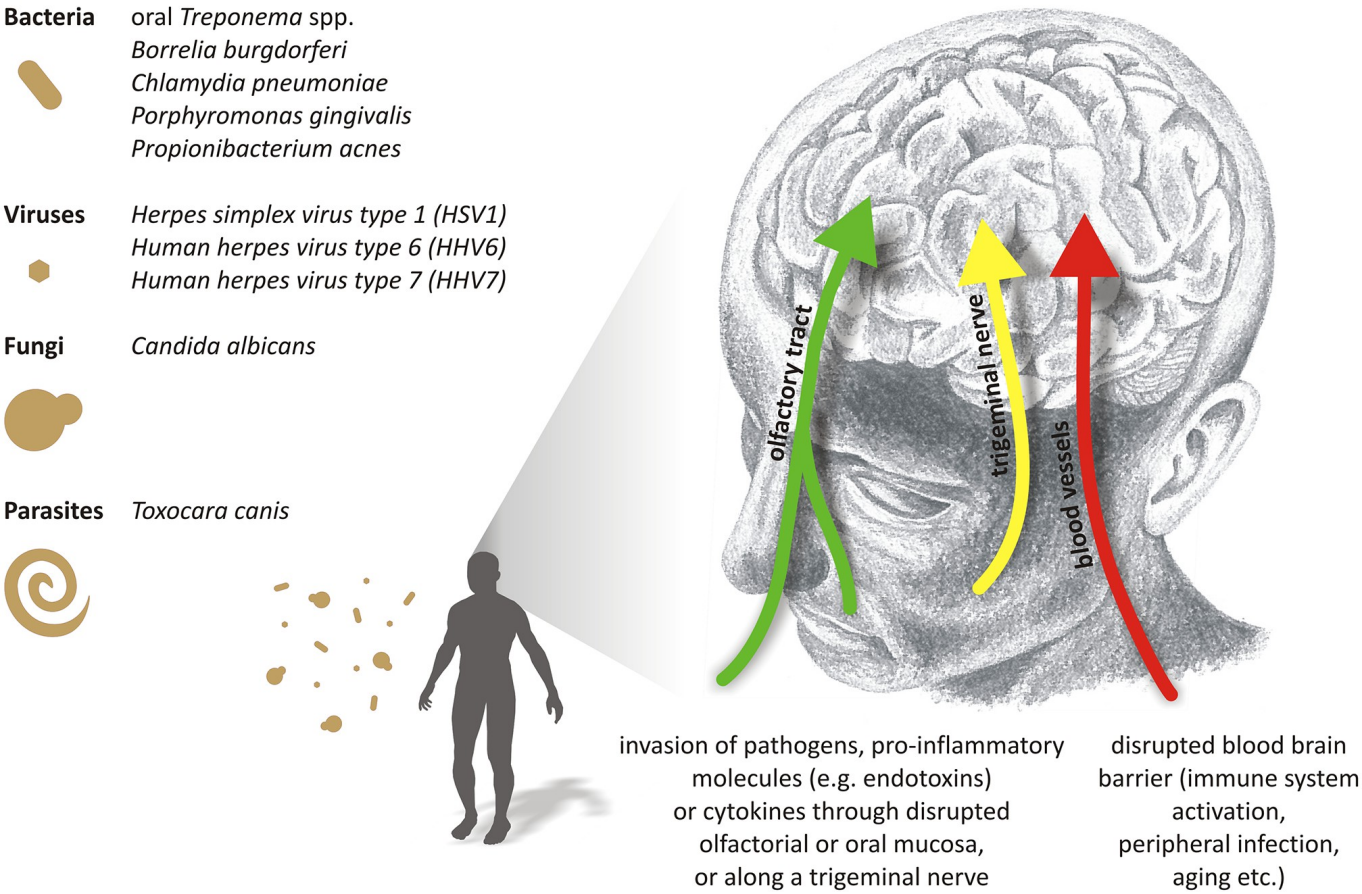
Pathogens possibly associated with AD pathology and suggested ways of their entry into the brain. On the left, the most important pathogens associated with AD discussed in this review. Pathogens inhabiting nasal and oral cavity can migrate through disrupted mucosa and then along the olfactory nerve (e.g., *Chlamydia pneumoniae*; left green arrow) or the trigeminal nerve (e.g., periodontal *Treponema* species and *Herpes simplex virus type 1*; middle yellow arrow). Microbes from both oronasal area and the periphery can enter the bloodstream during transient bacteremias or candidemias (e.g., *Porphyromonas gingivalis*, *Candida albicans*) and then attack the brain through disrupted BBB (right red arrow). Parasites like *Toxocara canis* also enter the CNS from the blood vessels. AD, Alzheimer’s disease; BBB, blood–brain barrier; CNS, central nervous system.

We believe that no major breakthrough in sporadic AD therapy is conceivable without understanding the primary causes. Although over 40 genetic loci (related to APP/tau processing, immune response, or other cellular processes) have been linked to the AD risk in genome-wide association studies [41], it is reasonable to think that the primary trigger comes from the outside. In the following sections, we discuss the existing evidence linking AD with specific groups of pathogens, namely bacteria, viruses, fungi, and parasites (see the list of pathogens the most discussed here in the context of AD, and possible ways of their entry into the brain, in Fig 2).

## Infectious origin of Alzheimer’s disease as an emerging field

### Bacteria and Alzheimer’s disease

The infectious hypothesis of AD, positing external pathogen as its primary cause, is hardly new. When AD was first described by Alois Alzheimer and Oskar Fischer in 1907 [42], the discoverers noticed similarities between AD and neurosyphilis, implying bacterial origin [43]. Infection of the CNS by bacteria *Treponema pallidum* in neurosyphilis causes microgliosis, cortical atrophy, amyloid plaques, and tauopathy [44]. Other spirochetes (such as *Borrelia*) are also known for their neurotropism [45]. Indeed, *Borrelia* spirochetes were repeatedly identified in the brain, cerebrospinal fluid, or blood of AD patients [43,44,46,47]. In some studies, the bacteria co-localized with amyloid plaques and neurofibrillary tangles in the brain tissue. The microbes were mostly identified as *Borrelia burgdorferi*, the causative agent of Lyme disease, a pathogen known for its ability to cause chronic CNS infections. Miklossy and colleagues demonstrated that exposure of human neurons and glia to *B*. *burgdorferi* cells *in vitro* leads to amyloid production and tau phosphorylation, showing the potential of spirochetes to facilitate AD-relevant processes [48].

Periodontal disease is a confirmed risk factor of AD, a predictor of faster disease onset, and is also known to accelerate the AD-like degenerative changes in Down syndrome [49–52]. Riviere and colleagues found that periodontal *Treponema* species are capable of invading the brain, and spirochetal infestation is more severe in AD patients [53]. We must critically note that those studies could not demonstrate the actual direction of causality, and several other attempts have failed to detect oral spirochetes in AD tissue [54–56]. Another member of oral microflora causing chronic periodontitis, *Porphyromonas gingivalis*, has been recently identified in the brain tissue of AD victims, and its ability to penetrate into the brain and stimulate Aβ production, neuroinflammation, and tau pathology was demonstrated in animal models and cell culture [57]. Of note, gingipains (proteases released by *P*. *gingivalis*) switch microglial activity toward neuroinflammatory response, leading to neuronal damage and impaired clearance of Aβ [58]. This opens the possibility that apart from effects of local brain infection, AD pathology could also be triggered indirectly by pathogen-derived molecules (virulence factors) produced elsewhere in the body. Although the clinical trial (NCT03823404) testing the gingipain inhibitor COR388 (atuzaginstat) failed [59], microbial toxigenic mechanism should not be generally rejected. For example, polysaccharide components of *P*. *gingivalis* extracellular capsule were also proposed to activate innate immunity and inflammation leading to AD-like pathology in a rat model [60].

Another line of research connects AD to infections by intracellular chlamydiae [61]. *Chlamydia (Chlamydophila) pneumoniae* is a prevalent respiratory pathogen, especially common in the elderly [62]. The infection may become systemic and enter the CNS [63,64]. Balin and colleagues reported *C*. *pneumoniae* in *post mortem* AD brain samples, with bacterial cells located inside pericytes, astroglia, and microglia [65]. Several later studies confirmed this association [66–68], although some did not [69–71]. Furthermore, the bacterial cells seem to be located in the areas primarily affected by the AD pathology, especially amyloid deposition [65,68,72]. The causal role of this pathogen in AD is strongly bolstered by murine model experiments, where *C*. *pneumoniae* is able to invade the brain through olfactory bulbs, triggering neuroinflammation, Aβ accumulation, and plaque formation, even in the absence of mutated APP [73–77]. Other common bacteria, such as *Helicobacter pylori* [78–81] and *Propionibacterium acnes* [82,83], have been implied in AD pathology; however, convincing evidence for the link is yet to be provided.

It seems that bacteria from very different taxonomical or ecological groups can all be relevant in the context of AD. Species capable of direct, chronic CNS infections seem to be particularly prominent, followed by bacteria characteristic for peripheral (oral) infections, which presumably invade the brain as opportunistic pathogens once the defense barrier is weakened by immunosenescence or chronic disease. However, brain invasion may not even be necessary as microbial virulence factors, such as lipopolysaccharide (produced by gram-negative bacteria) or gingipains (released by *Porphyromonas gingivalis*) can induce neuroinflammatory response, even in the absence of actual pathogen in the CNS [84].

### Herpes viruses in Alzheimer’s disease

Neurotropic viruses of the *Herpesviridae* family, especially the *Herpes simplex virus type 1* (HSV1), belong to the prime suspects, as they are highly prevalent, able to produce latent infections in neuronal cells, and preferentially infect brain structures typically affected in AD [85,86]. HSV1 infection initiates production of proinflammatory cytokines [87], which are also elevated in brains of AD patients [88]. In a mouse model of HSV1 recurrent infection, progressive accumulation of AD biomarkers (Aβ, hyperphosphorylated tau) was observed in the neocortex and hippocampus. It was triggered by repeated virus reactivations (typical for human herpes labialis) and correlated with cognitive deficits [89]. HSV1 infection could promote tau hyperphosphorylation via U_S_3 viral kinase activating protein kinase A [86]. Of note, AD incidence was highly correlated with reactivation of HSV seropositivity in humans as well [90]. It was also suggested that proinflammatory cytokines released during peripheral infections could stimulate neuroinflammation and HSV1 reactivation in the CNS, leading to AD development [86].

The presence of HSV1 was confirmed in various areas of AD brains [91,92], but not all AD patients seem to harbor the virus [93]. Moreover, HSV1 is not sufficient to cause AD by itself [91]. A strong risk emerges only in combination of latent infection with APOE4 [94,95]. APOE4 might facilitate entry of HSV1 virus into the brain or allow more efficient spread and replication of the virus in the brain. Both the virus and APOE protein compete for the same binding molecule, heparan sulfate proteoglycan, before entering the cell through specific receptors. The weaker binding of APOE4 makes it a weaker competitor, relative to the other APOE isoforms [86]. This is supported by the findings from APOE-transgenic mice, where the level of latent HSV1 DNA in the brain was much higher in mice expressing the human APOE4 compared to mice carrying the APOE3 allele [96,97].

*In vitro* observations confirmed that HSV1 infection increased intracellular Aβ levels [98] and activity of BACE1, the enzyme cleaving Aβ from APP [99]. It also induced AD-specific phosphorylation of tau protein [100], and neurite damage due to alterations in microtubule dynamics, followed by neuronal death [101]. In addition, HSV1 caused synaptic dysfunction in cell culture of cortical neurons [102] that is also one of the major hallmarks of AD [103]. Recent work by Cairns and colleagues used 3D cerebral organoids derived from human-induced neural stem cells to study the response to low-level infection by HSV1. The model replicates many AD hallmarks that could be prevented by timely application of valacyclovir [104].

The risk of senile dementia is higher in people infected with HSV. Conversely, anti-herpetic medications cause a dramatic decrease in the number of individuals who later develop dementia [105]. Furthermore, Wozniak and his colleagues found a great beneficial effect of antiviral agents on the levels of Aβ and abnormally phosphorylated tau protein in HSV1-infected cell cultures [106–109]. The tested antiviral drugs (e.g., acyclovir) inhibited HSV1 DNA replication that led to decrease in hyperphosphorylated tau accumulation [108]. Valacyclovir is a pro-drug that is converted by viral enzymes to acyclovir, incorporating into viral DNA and inhibiting viral DNA polymerase activity [110]. For nearly 2 decades, valacyclovir has been the most widely used antiviral treatment of peripheral HSV1 (herpes labialis), HSV2 (genital herpes), and some other viral infections, with a good CNS penetration and a great safety profile. Currently, valacyclovir proceeded to be tested in clinical trials (NCT03282916; NCT02997982) as the first antiviral drug with the potential to treat AD [110,111]. Another clinical study employed combined antiviral therapy by apovir (pleconaril and ribavirin) in AD patients [112]. While some of the results were mildly encouraging, the tolerability of the treatment was low and the drop-out rate high, making the study inconclusive. So far, antiviral therapy has brought promising results in possible reduction of the risk for development of clinical HSV1-associated AD [113]. Therefore, specific anti-herpetic treatment, in combination with anti-inflammatory treatment, might represent a promising therapeutic strategy to prevent AD occurrence in people without any clinical signs, but further confirmation of its efficiency is required. The evidence for the effect of antiviral agents in patients already suffering from the disease is missing and more studies in this field are necessary [114].

Besides HSV1, other herpes viruses have also been linked to AD. *Human herpes virus type 6* (HHV6) has been found in a higher proportion of AD patient brains in comparison to age-matched normal brains [115]. The risk was not associated with APOE4, but rather modulated by genes responsible for the NK-cell immune response [116]. HHV6 (and *Epstein–Barr virus*) positivity has been found to increase the risk of conversion to AD [117].

Several recent studies have also pointed at the *Varicella zoster virus*, responsible for herpes zoster, showing both its increased prevalence in AD patients and benefits of antiviral therapy and vaccination [118–120]. Cairns and colleagues have demonstrated *in vitro* that the effect of *Varicella zoster virus* is likely indirect. The virus elicits neither amyloid nor tau pathology, although it induces gliosis and neuroinflammation. However, it can reactivate HSV1 in quiescently infected cells, which then triggers both Aβ and hyperphosphorylated tau production [121]. Infection by *Cytomegalovirus*, another member of *Herpesviridae*, is positively correlated with clinical markers of AD [122]. Interestingly, it seems that the concurrent presence of antibodies against both *Cytomegalovirus* and HSV1, rather than *Cytomegalovirus* or HSV1 alone, is a significant AD risk factor for future AD development [123]. This may point at interaction between the viruses, similar to the one found for *Varicella zoster virus* by [121]. Caruso and colleagues have hypothesized that *Cytomegalovirus* infection induces a general pro-inflammatory state, which might be beneficial for younger individuals as it protects them against other infections, but becomes deleterious in the elderly, leading to chronic/latent inflammation and increasing their morbidity and eventual mortality [124].

*Human immunodeficiency virus* (HIV)-infected patients develop a form of dementia accompanied by increased Aβ production and plaque formation (reviewed by [125]). The presence of APOE4 allele was found to facilitate its progress [126,127], similarly to HSV1 infection. However, very little is known so far about the role of non-herpetic viruses (such as HIV) in AD pathology, and further investigation is required.

### Fungi and eukaryotic parasites in Alzheimer’s disease pathology

Besides bacteria and viruses, fungi and eukaryotic parasites have been implied in AD pathology. Wu and colleagues have recently demonstrated that a common pathogenic yeast *Candida albicans* crosses the mouse blood–brain barrier (BBB). Yeast aggregates in the brain induce local inflammation with microglial activation, production of inflammatory cytokines, and elevated production of APP and Aβ [128]. As for human samples, pathogenic yeasts or even other fungi (soil or phytopathogenic species) were proposed to be present in *post mortem* AD brains [93,129–134]. However, the studies included only a low number of samples, the authors admit unspecific binding of antibodies used for immunohistochemical detection, and environmental contamination by exogenous DNA cannot be fully excluded. Additionally, some of the signals obtained from next-generation sequencing could stem from off-target amplification in low biomass samples leading to false-positive results [135]. We suggest that these results should be further validated in independent studies prior to making strong conclusions.

The role of eukaryotic parasites in AD etiology/pathology has been rather neglected although there are many species affecting the human CNS [136]. Of them, *Toxocara canis* (a dog roundworm) and *Toxoplasma gondii* (an intracellular protist) have sparked special interest as they are worldwide distributed and cause chronic infections in humans. Cerebral toxocarosis is caused by the migration of *T*. *canis* larvae into the CNS. While the disease is hardly traceable in humans, the experimental mouse model is available and well characterized. Besides neuroinflammation [137,138] and neurobehavioral changes [139] observed in infected mice, 2 specific features were proposed to be of particular importance regarding AD pathology. First, it is increased production and progressive accumulation of APP, Aβ, and phosphorylated tau in mouse brains [140,141]. While APP localizes mostly to axons, indicating axonal injury [142,143], the distribution of Aβ has not been examined. Hence, it is unclear whether Aβ concentrates around the migrating parasites and forms oligomers. Second, large amounts of transforming growth factor-beta (TGF-β) precursor, positively correlating with the infection dose, are produced in infected mouse brains [140]. Except for TGF-β immunosuppressive and neuroprotective functions [144], it was shown to promote amyloidogenesis both *in vitro* and *in vivo* [145,146]. Based on these hints, Fan and colleagues proposed a possible role of chronic neurotoxocarosis in the initiation of AD-like pathology or even AD itself [147]. Unfortunately, experimental data testing this intriguing hypothesis (especially in long-term settings) are not available.

*Toxoplasma gondii* causes chronic infections characterized by the life-long presence of cysts in various tissues, including the CNS. In immunocompetent hosts, the infection is clinically asymptomatic, but behavioral alterations have been linked with latent toxoplasmosis [148,149]. While Kusbeci and colleagues reported higher *T*. *gondii* seropositivity among AD patients [150], other studies failed to confirm toxoplasmosis as a risk factor for AD [151,152] or general cognitive decline [153]. Accordingly, meta-analyses also reported only marginal association between toxoplasmosis and AD [154,155]. *T*. *gondii*-infected mice prone to AD pathology exhibited reduced Aβ plaques and milder learning and memory deficits, likely thanks to increased production of anti-inflammatory cytokines (IL-10, TGF-β) and improved clearance of Aβ [156,157]. Suppression of the inflammatory and Aβ response of the host is probably a part of the immune evasion strategy of the parasite. Correspondingly, reduced Aβ deposition correlated with remarkably higher parasite burden and persistence of the infection [158]. This supports the view that Aβ has protective antimicrobial functions (it can even accumulate around *T*. *gondii* cysts; [159]), and its depletion facilitates spreading of the infection. Interestingly, this effect is strain specific, and *T*. *gondii* strains that cannot induce clearance of Aβ plaques have lower parasite burden in AD mice [158] and trigger cortical neurodegeneration [160].

## Protective role of amyloid beta

The “antimicrobial protection hypothesis of AD” assumes that Aβ production, oligomerization, and fibrillization, in fact, represent an innate immunity of the brain aimed against pathogens [161,162]. It seems that Aβ exhibits many characteristics of the other known antimicrobial peptides [162,163]. *In vitro* studies combined with experiments on mice show a protective role of Aβ in case of bacterial, viral as well as fungal infections. Microbes or viruses were found to be associated with Aβ plaques [44,92]. Interestingly, experimental therapy targeting Aβ in AD patients results in increased infection incidence [23]. Recently, Alam and colleagues have shown a revolutionary finding that α-synuclein, a protein abnormally accumulated in the brains of patients suffering from Parkinson’s disease (and not previously suspected from immune actions), is an essential mediator of the immune responses and inflammation within the peritoneal cavity [164]. In comparison, Aβ physiological antimicrobial function and the accumulation within the brain contributing to AD development look markedly similar and are supported by independent observations from bacterial, viral, and fungal infections.

### Bacteria

An antimicrobial activity of Aβ, able to agglutinate and entrap bacteria, was assessed in several clinically relevant species [165,166], and in some cases, the bactericidal activity of Aβ was greater compared to the archetypal human antimicrobial peptide LL-37 [165]. Moreover, the antimicrobial activity was higher in temporal lobe homogenates from AD patients than in age-matched samples from non-AD subjects, and was correlated with Aβ levels [165]. Recently, the possibility has been raised that aggregation of Aβ, α-synuclein and perhaps other pathological proteins could be seeded by functional bacterial amyloid proteins, normally involved in cell adhesion and biofilm formation [167,168]. As some bacteria evade antimicrobial peptides and defuse them by producing trapping proteins that bind them with high affinity [169], triggering Aβ or α-synuclein aggregation on extracellular “seeds” could arguably serve the same purpose.

### Viruses

Studies on cell cultures revealed that especially Aβ1–42 oligomers are able to interact with HSV1 (and also HHV6), prevent their entry into the host cell and inhibit its replication [161,170]. In case of HSV1, Aβ binds directly to the viral particles, entrapping viruses in insoluble deposits through generation of Aβ fibrils on the viral surface [161]. Aβ probably interacts with viral coat proteins, since the replication of non-enveloped human adenovirus was not prevented by Aβ1–42 [170]. Soluble Aβ1–42 protected human neuronal–glial cell culture against HSV1 pathological effects with similar efficiency as the “classical” antiviral agent acyclovir [171]. In addition, transgenic mice producing human Aβ with mutations known to cause familial AD showed higher survival after application of lethal viral dose in comparison to wild-type mice. Moreover, administration of nonlethal viral dose to these mice led to Aβ deposits surrounding the viruses [161]. These observations were questioned by some subsequent studies [172,173]. However, this discrepancy could be explained by viral particle doses or viral strains/species used, which may differ in their sensitivity to Aβ. *In vitro* experiments showed the antimicrobial activity of Aβ also against enveloped *Influenza A virus*, with the Aβ1–42 being more efficient compared to Aβ1–40. Aβ inhibited viral replication, caused aggregation of viral particles and also facilitated uptake of viruses by leukocytes [174].

Aβ fibrils with bound nucleic acids (that can originate either from viral particles of damaged host cells) are highly immunogenic and elicit robust type I interferon secretion by adjacent microglia [175,176], which promotes their antiviral response. Activated microglia or other cells infiltrating the infected brain can also produce interferon-γ (IFN-γ) [177,178], which facilitates Aβ production (see above). This shows that Aβ is an integral part of the innate immune system antiviral response. The suggested positive feedback loop between Aβ and interferon signaling perhaps makes the system prone to self-perpetuating or excessive inflammatory responses.

### Fungi

In case of low-grade candidemia caused by *Candida albicans* in mice, Wu and colleagues observed glial granulomas in brain tissue [128]. They consisted of Aβ peptides accumulating around the yeast cells and accumulation of activated microglia and astroglia surrounding these aggregates. The authors have shown that Aβ binds to fungal cells, and although it does not destroy them directly, it stimulates phagocytic activity of microglia that also produce not yet identified antifungal soluble factors. Transgenic mice expressing human Aβ with familial AD mutations showed faster clearance of *C*. *albicans* from brains compared to wild-types and transgenic mice missing gene for APP [128].

It seems that Aβ, as an antimicrobial peptide, acts through 2 direct pathways: (1) binding of soluble Aβ oligomers to microbial surface carbohydrates that causes agglutination of microbes, entraps them into the fibrillar network, and so prevents them from invading the host cells and spreading within the tissues; and (2) fibrillization of Aβ oligomers on microbial surface to perturb microbial membranes [179]. Indirect effects involve the stimulation of other humoral or cellular components of the immune innate system such as microglia.

If Aβ serves as a brain protector against pathogens, why does the protective response overturn into a pathology which slowly and unavoidably kills the host? Aβ is a potent weapon against microorganisms, but also a double-edged sword that can damage and destroy neurons as well, unless tightly regulated. Butterfield and Lashuel suggest that Aβ peptides are able to create annular protofibrils that in higher concentrations disrupt cellular membranes [180]. For reasons that are not yet fully clear, this mechanism may turn against neuronal plasma membranes instead of the microbial ones and lead to destruction of the neurons. Killing of infected neurons by intrinsic protective peptides could be a meaningful response in the case of intracellular pathogens (such as viruses) able to inhibit cell apoptosis. Moreover, the chronic accumulation and fibrillization of Aβ may trigger the cascade leading to hyperphosphorylation of the tau protein, formation of neurofibrillary tangles [181], and finally, neuronal death, as described by the “amyloid cascade hypothesis” [29,161]. Disruption of the cerebral glymphatic system may be the key factor mediating the transition between the physiological and pathological role of Aβ [182]. If the clearance of Aβ, as well as hyperphosphorylated tau protein [183,184], becomes impaired, the molecules accumulate in the brain tissue and support chronic neuroinflammation. Whether hyperphosphorylated tau protein is also part of brain innate immunity has not been clarified so far.

Under normal conditions, BBB presumably prevents most pathogens from entering the brain at all and the innate response involving Aβ is therefore rarely activated. Even then, it usually deals with the infection in a quick and constrained manner, causing only limited damage to neural cells. However, these “acceptable losses” might easily become excessive during chronic infections of the CNS, or when the defensive response fails to be terminated, leading to progressive neurodegeneration apparent in AD. This also explains why it is typically seen in an aging organism, where the peripheral defenses are weakened, the pathogen load is typically high, and the senescent immune system is inclined towards prolonged inflammatory responses.

## Reverse transcription and genome insertion of APP mRNA: Friend or foe?

**Fig 3:**
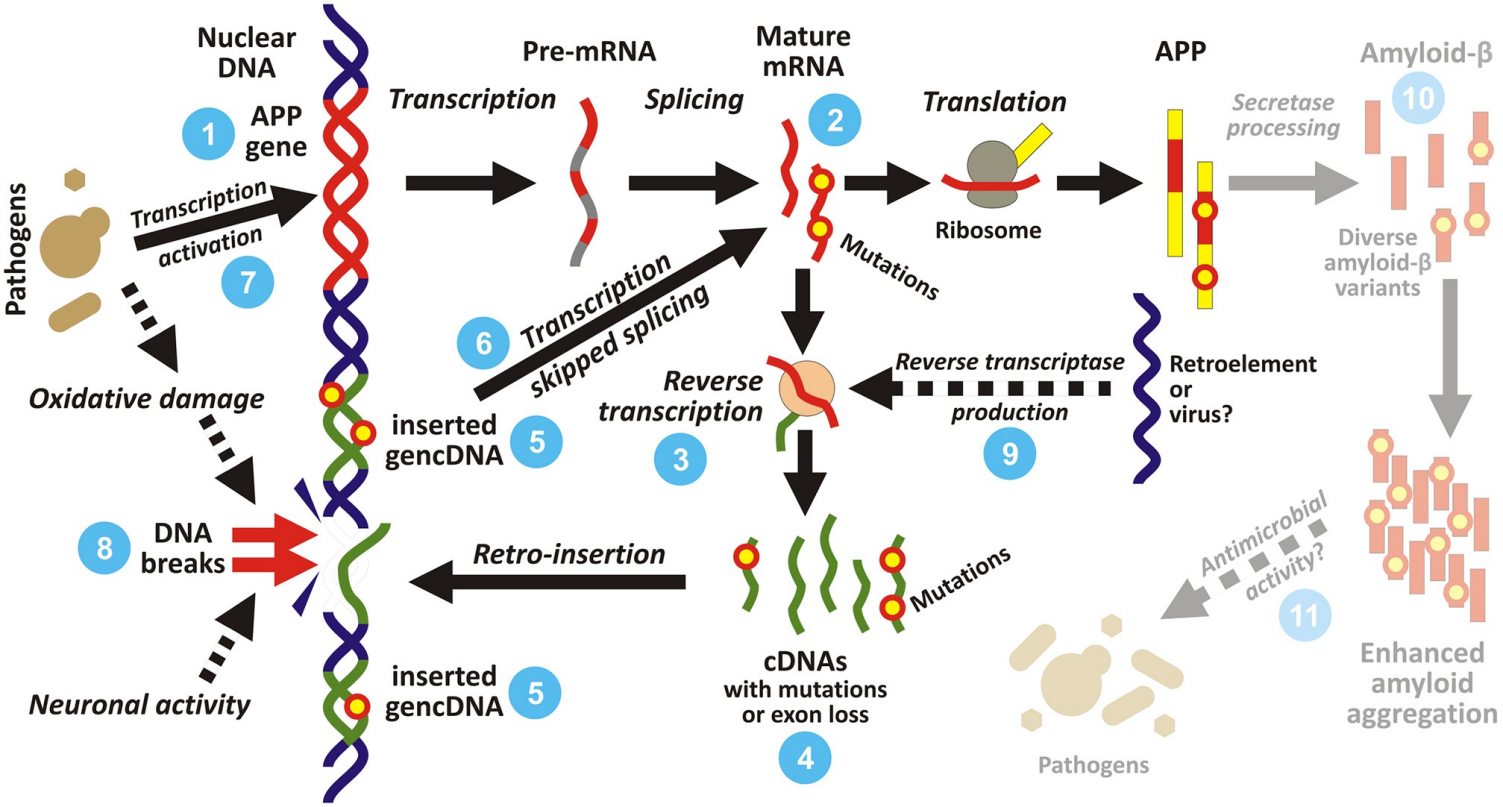
Somatic *APP* gene recombination. In human neurons, additional copies of the *APP* gene are created by the process of somatic recombination. The original *APP* gene (**1**) is undergoing standard transcription and splicing. Mature APP mRNAs (**2**) can undergo the process of reverse transcription (**3**) to complementary DNAs (cDNAs; **4**), which can be retro-inserted into genomic DNA as genome-inserted complementary DNAs (gencDNAs; **5**). The resulting additional copies of *APP* gene lack introns and often bear point mutations or exon deletions as both regular and reverse transcription are error prone, but can still be transcribed and translated (**6**). Moreover, the resulting mRNAs may undergo repeated cycles of retro-insertion, leading to accumulation of errors. We propose that cerebral infection provides some of the necessary ingredients for this mechanism, namely activation of *APP* expression (**7**), oxidative damage leading to DNA breaks (**8**), and a source of reverse transcriptase (**9**). Enhanced APP production by gene multiplication, and even the production of aggregation-prone mutant forms of amyloid beta (**10**) might act as an antimicrobial defensive mechanism (**11**). However, it is also harmful to the tissue itself, and as the mutant gencDNA insertion is irreversible, it may easily open the door to progressive neurodegeneration. APP, amyloid precursor protein; gencDNA, genome-inserted complementary DNA.

In recent years, the phenomena of somatic genomic mosaicism and somatic gene recombination in neuronal cells have been implied in the origin of neurodegenerative disorders, particularly AD, although some of the findings are disputed (reviewed in [185,186]). Bushman and colleagues [187] have observed that neurons from the AD brain cortex have increased DNA content by 8% relative to neurons from age-matched control brains. This increase, however, was not seen in cerebellar neurons and somatic cells of the same AD patients. In addition, the study reported increased copy numbers of the *APP* gene in cortical neurons from the AD brains relative to non-AD control tissue. A subsequent study by the same team [188] elaborated this observation and found neuron-specific insertions of DNA copies, derived from APP mRNA (termed genome-inserted complementary DNAs, gencDNAs), into the nuclear genome (Fig 3). These gencDNAs lack introns and often bear point mutations or exon deletions as both regular and reverse transcription is error prone, but can still be transcribed and translated, producing abnormal protein products. Moreover, the resulting mRNAs may undergo repeated cycles of retro-insertion, leading to cumulative errors. In AD patients, *APP* gencDNAs are not only more numerous, but also often bear harmful mutations usually linked to familial AD [188].

The authors suggest that the somatic gene recombination of *APP* may induce sporadic AD via mechanisms partly analogous to familial AD. Whereas in familial AD, all neurons exhibit the disease-linked genotype, sporadic AD would be initiated by a subpopulation of neurons featuring somatic mutations with similar effects (overexpression of normal or mutated APP) [189]. Abnormal protein products of *APP* gencDNAs might be especially toxic, initiating amyloid aggregation or other pathological mechanisms [189]. As the insertions and mutations presumably accumulate during the lifetime, they may explain the age-dependent and seemingly random occurrence of sporadic AD.

Creation of gencDNA requires several ingredients: expression of the gene, reverse transcription of its mRNA into DNA, and breaks in the genomic DNA strands allowing integration of the gencDNA. The source of reverse transcriptase activity, in particular, is completely unknown, as humans lack intrinsic reverse transcriptase. We propose that cerebral infection may provide the necessary conditions. *APP* transcription is up-regulated as a consequence of infections, while DNA strand breaks are induced during neuroinflammation and oxidative stress, and are linked with AD pathology from early stages onwards [190–192]. The last crucial mechanism, reverse transcriptase, might be provided by external retroviral infection, human endogenous retrovirus, or a retrotransposon. Retrotransposons and human endogenous retroviruses are activated under specific conditions, including herpetic infections [193], neuroinflammation, and aging [194,195]. Indeed, activation of retroviruses and transposable elements has been linked to aging-related neurodegenerative disorders [194,196]. Lee and colleagues suggest that patients receiving reverse transcriptase inhibitors may show lower AD risk, but the evidence for this correlation seems inconclusive [188] and requires further research.

We may speculate about the purpose of the gencDNA-creating mechanism (Fig 3). The discoverers consider that elevated gene dosage, leading to increased gene transcription with bypassing splicing, is beneficial for a cell when it needs a higher amount of APP [188]. If Aβ acts as an antimicrobial peptide, it is tempting to speculate that even the elevated mutagenesis might have its benefits, similarly to suggested enhancement of the function of some known antimicrobial peptides by polymorphism or posttranslational modifications [162]; however, this needs to be experimentally tested. Indeed, the *APP* mutations leading to familial AD may increase the antimicrobial activity of Aβ. However, it is also harmful to the tissue itself, and as the mutant gencDNA insertion is irreversible, it may easily open the door to progressive neurodegeneration.

There are still a lot of unknowns surrounding the somatic gene recombination mechanisms in AD. The very existence of gencDNAs has been questioned as an experimental artifact resulting from sample contamination [197], although the major part of the claims by [188] seem to be valid [198] and independently verified [199]. The physiological role of somatic gene recombination, if any, is unknown. The total number and diversity of gencDNAs might be underestimated by the current detection techniques [189]. It is not known whether the process is *APP* specific or also includes other genes. The evidence for gencDNA of the presenilin gene has been negative so far [188]. Somatic point mutations in AD brain tissue have been found in the genes contributing to tau hyperphosphorylation, although only in 27% of the examined brains [199]. Mutations in genes related to neuroprotection, cytoskeleton remodeling, autism, and intellectual disabilities have also been found to be more abundant in AD brains [200]. Interestingly, copy number variants of the gene for α-synuclein have been found in synucleinopathies [201], suggesting that the same mechanism may be common to this class of disorders as well.

## Summary and concluding remarks

The literature evidence strongly indicates that Aβ production, apart from being hallmark of AD, is also an integral part of the brain innate immune response. Therefore, we may view AD as a pathological consequence of brain immunity activation, and search for its trigger, be it infection or another kind of insult. The existing body of evidence strongly suggests there is no specific “Alzheimer’s germ” (see alzgerm.org). Most probably, any microbe capable of entering the brain and causing a chronic infection could be a culprit, explaining the mixed results of many previous studies focused exclusively on certain classes or species of organisms. Some known or suspected AD risk factors may facilitate brain infection by promoting pathogen spread, compromising the BBB or peripheral immune system function. The most prominent risk factor of AD is aging. This fits the general picture well, as senescence of the immune system compromises its ability to suppress pathogens, and at the same time, makes it susceptible to prolonged and maladaptive inflammatory reactions.

**Fig 4:**
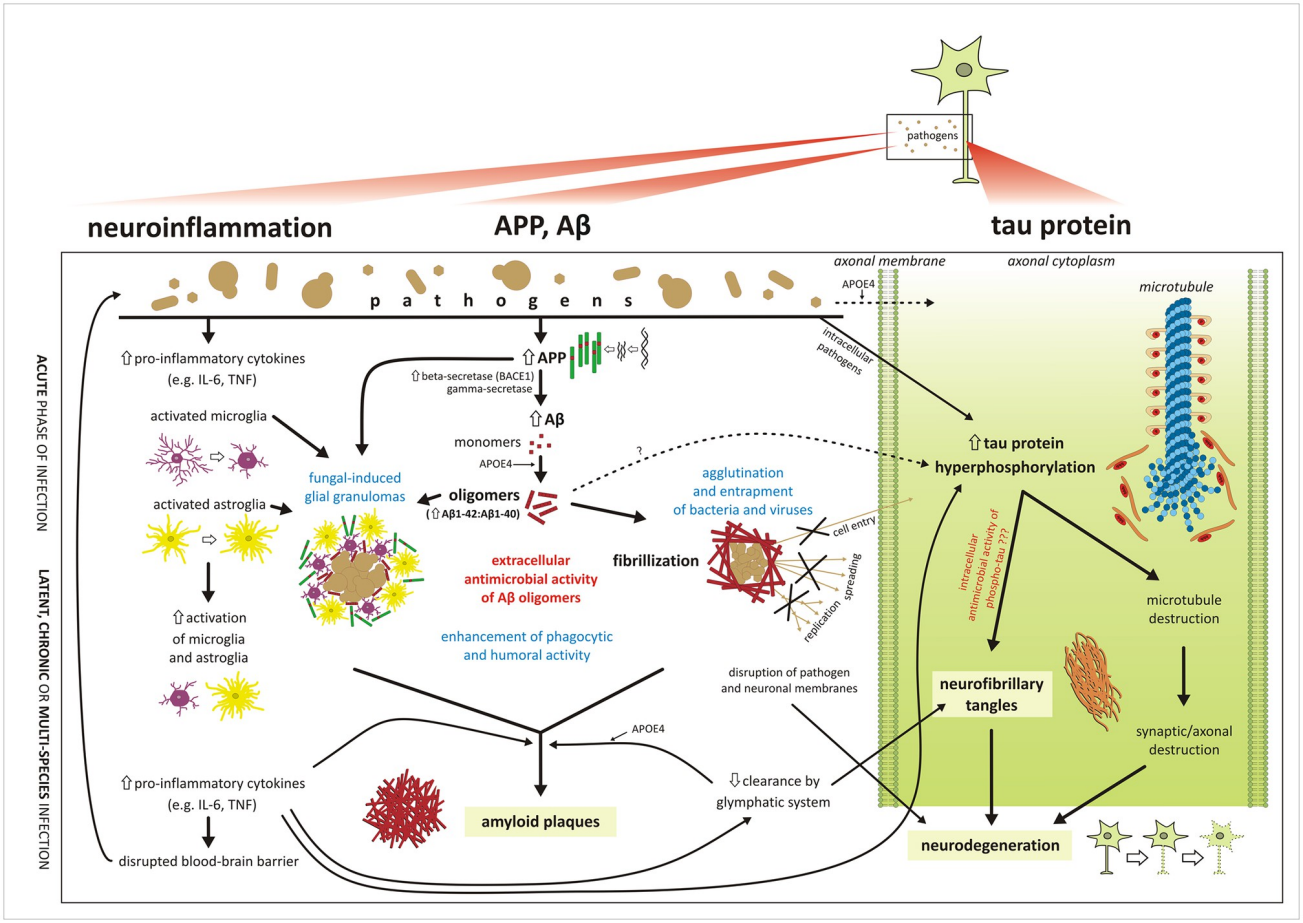
Schematic illustrating the infectious hypothesis of AD. The scheme follows the 3 main domains of AD: neuroinflammation, amyloid pathology, and tau protein pathology, progressing from the top to the bottom. Pathogens (bacteria, viruses, and/or fungi) entering the CNS activate the innate immunity. If the inflammatory process becomes chronic, the BBB is disrupted, facilitating entrance of further pathogens into the brain. Expression of APP and its cleavage to Aβ is enhanced in reaction to pathogens. Aβ monomers cluster into oligomers that can entrap various pathogens (agglutination or granuloma formation), which prevents them from entering into neurons, spreading or replicating, and facilitates their destruction by microglia. Aβ fibrils can directly disrupt pathogens’ membranes; however, they may attack neuronal membranes as well. During chronic or latent infections, Aβ fragments and fibrils accumulate, and finally create insoluble amyloid plaques. Tau pathology is stimulated by Aβ and neuroinflammation by several pathways and might be directly triggered by intracellular pathogens. An interesting question is whether tau protein could also serve as an intracellular antimicrobial peptide, similarly to Aβ oligomers. Accumulation of Aβ and tau is aggravated by impaired clearance activity of the brain glymphatic system, which is also compromised by neuroinflammatory processes. APOE4, the strongest known genetic risk factor of sporadic AD, apparently affects oligomerization or clearance of Aβ and facilitates entry of some pathogens (e.g., HSV1) into the cell. Aβ, amyloid beta; AD, Alzheimer’s disease; APOE4, apolipoprotein E4 allele; APP, amyloid precursor protein; BBB, blood–brain barrier; CNS, central nervous system; HSV1, Herpes simplex virus type 1; IL-6, interleukin-6; TNF, tumor necrosis factor.

When the immune system is unable to destroy or suppress the pathogen, chronic inflammation causes progressive damage to the cerebral tissue; runaway degenerative processes may ensue, including the amyloid cascade as implied by the classical hypothesis (Fig 4). Self-perpetuating inflammation with amyloid overproduction might then persist even after elimination of the invading pathogen.

Both chronic inflammation on the background of immunosenescence and advanced age weaken the BBB and mucosal barriers, offering opportunities for pathogens from the periphery to invade the brain, and coinfections may be a rule rather than exception. Future studies should encompass as broad a spectrum of pathogens as possible, since the contributions of different microbial species to the initiation and progression of AD are largely unexplored.

AD is most probably not a classical infectious disease in the sense of Koch’s postulates, as it lacks a specific infectious agent, and pathogen invasion may not be the exclusive trigger. This certainly poses a considerable challenge for further research. Perhaps, the major hurdle is establishing the direction of causality: Do microbes trigger AD or does AD make the patients more susceptible to opportune infections? The only solid evidence for a causal role of microbes so far comes from cell cultures and animal models, and any such finding should be translated to human patients with due caution.

Despite the mounting evidence for the infectious hypothesis, there are also other known triggers leading to Aβ response, e.g., traumatic or vascular damage, not to speak of idiopathic overexpression of Aβ in familial AD or Down syndrome. Future studies should take these differences into account, as they could be reflected in drug sensitivity. Infectious theory of AD also provides clear directions for future research into novel therapeutic strategies. Prevention of infections by the relevant pathogens including meticulous oral hygiene should be the first option if applicable, together with emphasis on correct diagnosis and thorough management of chronically infected patients. In the future, targeted antimicrobial therapy could be applied to MCI or AD patients. Untimely administration of anti-Aβ agents might do more harm than benefit by disinhibiting eventual pathogens, but in cases where the infection has been resolved, anti-inflammatory and anti-Aβ agents may still provide clinical benefits. Improved diagnostic methods would be required for both early recognition of MCI patients and identification of the relevant cerebral pathogens. As most of the microbes implicated in AD are very proficient in resisting both the host immune response and the available treatments, finding novel therapeutics suppressing such persistent infections of the CNS seems to be the second necessary step in that direction. Doxycycline-rifampicin treatment has been already tried with moderate success in a clinical study [202], and penicillin, combined with supplemental drugs disrupting biofilms, was suggested against cerebral spirochetes [203]. Some other antimicrobial and antiviral candidate drugs were nominated [204].

Once we accept that the etiology of sporadic AD could be linked to prolonged innate immune response in the brain, triggered by pathogen invasion on the background of immunosenescence and age-related biological changes, individual pieces of evidence start to come together, linking all the known risk factors of sporadic AD and also explaining the gulf between animal model studies and clinical trials. We hope this new paradigm may open the way toward better understanding of AD and, ultimately, also provide the means to treat or prevent this fearsome disease.
